# Attitude change and increased confidence with management of chronic breathlessness following a health professional training workshop: a survey evaluation

**DOI:** 10.1186/s12909-020-02006-7

**Published:** 2020-03-30

**Authors:** Kylie N. Johnston, Mary Young, Debra Kay, Sara Booth, Anna Spathis, Marie T. Williams

**Affiliations:** 1 School of Health Sciences, Innovation, Implementation and Clinical Translation in Health (IIMPACT), University of South Australia, Adelaide, South Australia Australia; 2 Department of Thoracic Medicine, Central Adelaide Local Health Network, Adelaide, South Australia Australia; 3 Adelaide, South Australia Australia; 4 Cambridge Breathlessness Intervention Service, Cambridge University Hospitals NHS Foundation Trust, University of Cambridge, Cambridge, UK

**Keywords:** (3–10): chronic breathlessness, Education, Health professional

## Abstract

**Background:**

Clinicians and people living with chronic breathlessness have expressed a need to better understand and manage this symptom. The aim of this study was to evaluate a 3-day health professional training workshop on the practical management of chronic breathlessness.

**Methods:**

Workshop design and delivery were based on current understandings and clinical models of chronic breathlessness management, principles of transformative learning, and included sessions co-designed with people living with breathlessness. Registrants were invited to complete pre and post-workshop surveys. Pre and 1-week post-workshop online questionnaires assessed familiarity and confidence about workshop objectives (0[lowest]-10[highest] visual analogue scale), attitudes and practices regarding chronic breathlessness (agreement with statements on 5-point Likert scales). Post-workshop, participants were asked to describe implementation plans and anticipated barriers. Baseline familiarity and confidence were reported as mean (SD) and change examined with paired t-tests. Pre-post attitudes and practices were summarised by frequency/percentages and change examined non-parametrically (5-point Likert scale responses) or using a McNemar test of change (binary responses).

**Results:**

Forty-seven of 55 registrants joined the study; 39 completed both pre and post-workshop questionnaires (35 female; 87% clinicians; median 8 years working with people with chronic breathlessness). Post-workshop, greatest gains in confidence were demonstrated for describing biopsychosocial concepts unpinning chronic breathlessness (mean change confidence = 3.2 points; 95% CI 2.7 to 4.0, *p* < 0.001). Respondents significantly changed their belief toward agreement that people are able to rate their breathlessness intensity on a scale (60 to 81% agreement) although only a minority strongly agreed with this statement at both time points (pre 11%, post 22%). The largest shift in attitude was toward agreement (z statistic 3.74, *p* < 0.001, effect size *r* = 0.6) that a person’s experience of breathlessness should be used to guide treatment decisions (from 43 to 73% strong agreement). Participants’ belief that cognitive behavioural strategies are effective for relief of breathlessness changed further toward agreement after the workshop (81 to 100%, McNemar test chi- square = 5.14, *p* = 0.02).

**Conclusion:**

The focus of this training on biopsychosocial understandings of chronic breathlessness and involvement of people living with this symptom were valued. These features were identified as facilitators of change in fundamental attitudes and preparedness for practice.

## Background

Chronic breathlessness is a common and disabling symptom of many advanced diseases (heart, lung and cancer). Over the last 20 years, understanding of the neurophysiological mechanisms of chronic breathlessness has rapidly advanced [1]. When breathlessness is persistent despite diagnosis and optimal medical management [2], there are a range of pharmacological [3, 4] and non-pharmacological multidisciplinary strategies that can help people better manage breathlessness and improve quality of life [5, 6]. Health care provider beliefs and attitudes regarding symptoms are also vital as they can contribute to, or reduce disability [7]. Parallels between changes in how chronic pain is now perceived, assessed and managed, and chronic breathlessness have been highlighted [8]. While the education of health professionals has moved to incorporate pain neuroscience in line with established international pain curricula [9, 10], no such well-defined implementation pathways are yet in place for chronic breathlessness.

Health professionals [11, 12] and people living with chronic breathlessness [13] have expressed the need for education to better understand and manage chronic breathlessness. The Breathing, Thinking, Functioning (BTF) clinical model developed by Spathis et al. [14] is used within the Cambridge Breathlessness Intervention Service [15–17] to (1) engage patients in self management; (2) explain the multiple factors contributing to breathlessness and (3) facilitate clinician choice of non-pharmacological interventions. The BTF model has been used as an educative framework for teaching health care clinicians, consumers, and carers about breathlessness genesis and management [14]. Preliminary reports on delivery of a 2-h practical workshop (*n* = 40) using the BTF clinical model show an increase in participants’ confidence to manage breathlessness. Pre-workshop, 15% of participants rated their confidence at a level of “quite a bit” to “very well”, while 72% rated confidence at this level post-workshop [18].

To date, few published studies have explored the impact of educational interventions specific to chronic breathlessness for health professionals [19, 20]. Following an 8-day educational intervention (over 6 weeks in 2001) for breathlessness management, Froggatt and Walford [19] reported improvements in nurses’ (*n* = 12) familiarity, confidence and self-reported practice change. Similarly, after participation in a 2.5 day training program (over 3 months in 2013–14) on practical skills to support the breathless patient, Shaw et al. [20] reported improvements in confidence (of at least one step on a 4 point scale) in 21 of 25 participants (nurses, allied health professionals and healthcare assistants). While these suggest improved familiarity in attendees with relevant skills in assessment and promoting non-pharmacological strategies, recent reports indicate that challenges continue to be experienced by clinicians in talking about or managing chronic breathlessness with people under their care [21, 22]. Difficulties described by respiratory medicine trainees in initiating conversations about breathlessness include a perceived inability to provide a solution, or lack of knowledge about potential resources and thus giving a lower priority to discussing this symptom [21]. To support change in practice, fundamental changes are needed in attitude based on a reconfigured understanding of chronic breathlessness, insight into the impact of this symptom grounded in the experience of people with chronic breathlessness and further evidence-based resourcing about management opportunities.

The aim of this paper is to describe the nature and content of a purpose-developed health professional training workshop (Practical Management of Chronic Breathlessness) and to report evaluation of its immediate impact on participants’: (1) familiarity and confidence in the practical management of chronic breathlessness; (2) attitudes regarding chronic breathlessness assessment and management; and (3) planned behaviour change in clinical practice.

## Methods

### Design

We conducted a single group evaluation of a 3-day health professional training workshop on the practical management of chronic breathlessness, using repeated measures conducted pre- and post- workshop delivery. This study was approved by the University of South Australia Human Research Ethics Committee (Application ID 202195, approval date 20/5/2019) and was conducted between the approval date in May and July 2019, including delivery of the 3-day workshop in June 2019.

### Participants

Health professionals working with people who experience chronic breathlessness were eligible to participate in this workshop. Information about the training opportunity was distributed throughout Australia to state and national respiratory medicine and palliative care-focused organisations. All health professionals who registered for the training workshop were invited to participate in the evaluation.

### Health professional training workshop

The approach to development of this educational intervention was multifaceted, incorporating adult learning understandings [23], involvement of people living with breathlessness [24], and transformative learning theory (encouraging participants to critically reflect on and question their assumptions, beliefs and values [25]). Workshop content (Additional File 1) was based around the BTF clinical model [14] that incorporates neurophysiology of breathlessness, cycles of breathlessness-related cognition, function and behaviour and associated practical evidence-based intervention strategies.

*The aim of the workshop* was to develop knowledge, skills and confidence in the non-pharmacological management of people living with chronic breathlessness. Workshop objectives were developed prospectively (Table 1). The workshop aims and objectives were included in the information distributed about the training opportunity. Registration was not limited by professional discipline, but as the workshop aim focused on non-pharmacological management, we anticipated that fewer medical (than nursing or allied health) professionals might register.

**Table 1:** Objectives of the workshop

After completion of the workshop, participants will be able to:	
1.	Describe current biopsychosocial concepts underpinning the experience of chronic breathlessness.
2.	Undertake a person-centred assessment of the breathlessness experience and symptom needs of a person living with or caring for a person living with this symptom.
3.	Explain chronic breathlessness to a person living with or caring for a person living this symptom using jargon and value - free language.
4.	Describe and critique a range of instruments appropriate for assessment and monitoring chronic breathlessness.
5.	Describe clinical models to inform assessment and choice of management strategies.
6.	Demonstrate practical, evidence-based non-pharmacological management strategies for chronic breathlessness.
7.	Reflect upon own beliefs and expectations of chronic breathlessness and how these may contribute to the client experience and management.
8.	Identify resources for understanding and managing chronic breathlessness.

### Consumer participation

Development of this workshop was informed by a team that included a consumer researcher and two consumer representatives living with chronic breathlessness. All team members contributed to the design and delivery of a key aspect of this workshop: the “Conversations about breathlessness” sessions. The intent of these conversation sessions was to prioritise individual narratives of breathlessness experiences and prior interactions with health professionals. For these sessions, expressions of interest to participate were extended to people living with breathlessness from a local support group and physiotherapy practice. Four people living with chronic breathlessness volunteered to participate in conversation sessions as a result of this process.

*Workshop delivery* made use of a variety of interactive strategies (Additional File 1). Presentations of current thinking and research evidence on mechanisms, assessment, and non-pharmacological management of chronic breathlessness [1, 2, 6, 12, 14–17] were combined with practical experience in and critical reflection on assessment tools, explaining breathlessness to a person with this symptom,

### Questionnaires

Baseline (after registration) and immediate post-course (1 week) questionnaires were created using the electronic platform Survey Monkey and sent by email to participants, followed by one email reminder if not completed within a week. All participants provided informed, written consent prior to questionnaire completion. Components included in the questionnaire at each time point are summarised in Table 2 (complete questionnaires in Additional File 2).

**Table 2:** Components of the evaluation questionnaires

Component	Pre-course	1 week post-course
Demographic information	✓	
Familiarity and confidence in workshop objectives	✓	✓
Attitudes and practices	✓	✓
Needs analysis (additional to workshop objectives)	✓	
Course feedback		✓
Implementation/impact planning		✓

*Familiarity and confidence* regarding key aspects of the workshop content were assessed using questions based on those of Froggatt et al. [19], tailored to the eight specific objectives of this workshop. Participants rated their familiarity (0 = very unfamiliar, 10 = very familiar) and confidence (0 = not at all confident, 10 = very confident) concerning aspects of the management of chronic breathlessness using a 0–10 visual analogue scale (VAS).

*Attitudes* were examined using questions modified from a previous survey of health professional attitudes toward assessment and management of breathlessness [26] (used and modified with permission from the authors). While the original survey assessed hospital doctors’ attitudes toward dyspnoea assessment and management in patients with acute cardiopulmonary diseases [26], in our study modifications were made to align the questionnaire wording with our focus on chronic breathlessness. For example, an original survey statement was: “The patient’s experience of dyspnea should be used to guide treatment decisions independent of objective measures such as respiratory rate and oxygen saturation”. This statement was revised to: “The *person’s* experience of *chronic breathlessness* should be used to guide treatment decisions independent of objective measures such as respiratory rate and oxygen saturation”, to improve suitability of the statement for managing chronic breathlessness in a variety of settings. For these items participants read statements about the assessment and management of chronic breathlessness and were invited to indicate their agreement/disagreement on a 5-point Likert scale (1 = strongly disagree to 5 = strongly agree, 9 items) or to select one or more statements that most applied to them from a list (4 items).

*Impact planning* was assessed using one multiple choice response question (How do you plan to use information and skills gained from this workshop?) and one open response question.

### Questionnaire piloting

Electronic questionnaires were piloted on a convenience sample of the target audience (*n* = 3) to determine user acceptability, face validity, comprehensiveness and estimate completion time. All pilot users completed the survey in less than 10 min. No major revisions were suggested but minor changes to improve question clarity and correct technical errors were made prior to survey dissemination. Those who piloted the questionnaire did not participate in the main study.

### Data analysis

Response rate was calculated as the number of participants who joined the evaluation study as a percentage of all registered participants. Demographic data were reported descriptively (frequency, mean and standard deviation [SD], median and interquartile range [IQR] for non-normal distributions) to describe the respondents as a group. Characteristics of participants who did and did not complete a post-workshop survey were compared using t-test (for normally distributed continuous variables)/chi squared tests (categorical variables)/non-parametric tests (non-normal distributions). Internal consistency of the items used to assess familiarity (eight items), confidence (eight items) and attitudes (nine Likert-scale questions) were examined by calculating Cronbach’s alpha for each construct (baseline responses). Pre-post VAS scores for individual questions regarding familiarity and confidence were reported as mean (SD) and change examined with paired t-tests (distribution normality confirmed), with Cohen’s d reported to indicate effect size. Pre-post responses to Likert-style questions about attitudes were summaried by counts and percentages in each response category (with graphical display of percentage of respondents who somewhat/strongly agree with each statement) and change over time in 5-point Likert scale responses examined using Wilcoxon signed rank tests and effect size reported using Cohen’s r [27]. Responses to non Likert-style attitude questions were summarised descriptively and McNemar tests used to explore change in pre-post workshop attitudes (for binary responses). Free text responses to most/least helpful course features, suggested changes, other comments and planned impact on practice were summarised descriptively.

## Results

Fifty-five registrants attended the workshop in June 2019. Of these, 47 joined the evaluation study and submitted the pre-workshop survey providing baseline data (pre-course survey response rate: 47/55 = 85%). Thirty-nine of these study participants also submitted the 1-week post-workshop survey thus matched pre-post data were available in 39/47 (83%) participants (39/55 or 71% of all workshop registrants). Two additional registrants completed a post-course survey (post-course survey response rate: 41/55 = 75%).

### Characteristics of study participants

Participants completing the baseline survey (*n* = 47, Table 3) were predominantly female (91%), working in clinical roles involving direct client management (87%), from nursing and allied health disciplines. Respondents reported extensive prior employment in working with people with chronic breathlessness (median years 7, IQR 11, range 1–32 years), and currency of experience with over 80% of participants conversing about chronic breathlessness at least once a week or more frequently in the previous 3 months. No differences were observed between respondents that did (*n* = 39) or did not complete (*n* = 8) post-workshop surveys in baseline characteristics (age, gender, professional role, discipline, self-rated experience and expertise, frequency of conversations about breathlessness, *p* > 0.05 all comparisons).

**Table 3:** Characteristics of study participants

Characteristic	Submitted baseline survey, *n* = 47 n (%)	Submitted baseline and post-surveys, *n* = 39 n (%)
Gender (female/male)	43/4 (91/9)	35/4 (90/10)
Age (years)	40.7 (11.0)^*^	40.5 (10.5)^*^
Professional role in the last 6 months		
Mainly clinical (direct patient management)	41 (87.2)	34 (87.2)
Hospital (public)	24 (51.1)	21 (53.8)
Hospital (private)	1 (2.1)	1 (2.6)
Primary/Intermediate Care	4 (8.5)	2 (5.1)
Community-based (public)	5 (10.6)	4 (10.3)
Community-based (non-government)	1 (2.1)	1 (2.6)
Community-based (private practice)	3 (6.4)	2 (5.1)
Community-based (palliative care)	3 (6.4)	3 (7.7)
Mainly non-clinical (e.g. academic/ research)	6 (12.8)	5 (12.8)
University teaching	3 (6.4)	2 (5.1)
University full-time research	2 (4.3)	2 (5.1)
University full-time post-grad coursework	1 (2.1)	1 (2.6)
Professional discipline		
Allied Health Assistance	1 (2.1)	1 (2.6)
Nursing	12 (25.5)	10 (25.6)
Occupational Therapy	2 (4.2)	1 (5.1)
Physiotherapy	32 (68.1)	27 (69.2)
How many years have you been practicing with people with chronic breathlessness? (years)	7 [11]^#^	8 [10]^#^
In the past 3 months, how often have you had a conversation about chronic breathlessness?		
At least once a day	15 (31.9)	12 (30.8)
At least once a week	24 (51.1)	20 (51.3)
At least once a month	5 (10.6)	5 (12.8)
Not at all	0	0
No direct clinical contact with people with chronic breathlessness	3 (6.4)	2 (5.1)
How would you rate your expertise in chronic breathlessness? (0–10 VAS)	5.4 (1.7)^*^	5.5 (1.5)^*^

*mean [SD]; ^#^median [IQR]; *VAS* visual analogue scale

### Baseline familiarity, confidence and attitudes in breathlessness management

#### Familiarity and confidence

**Fig. 1:**
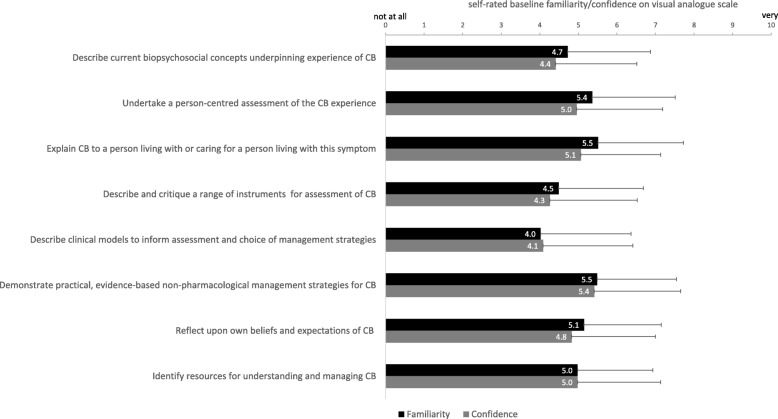
Baseline self-rated familiarity and confidence of participants (*n* = 47) with the course objectives. Visual analogue scale (VAS) anchors for familiarity self-ratings were 0 = very unfamiliar, 10 = very familiar. VAS anchors for confidence self-ratings were 0 = not at all confident, 10 = very confident). Bars indicate mean (solid bar) and standard deviation (error bar). CB = chronic breathlessness

Baseline responses (*n* = 47) are presented in Fig. 1. Internal consistency of both familiarity and confidence were acceptable (Cronbach’s alpha =0.931 and 0.957 respectively). Mean scores for familiarity and confidence for specific course objectives generally reflected the mid-way point of the 0–10 scale with the exception of describing current biopsychosocial concepts underpinning the experience of breathlessness, describing and critiquing instruments to assess breathlessness, and describing clinical models to inform assessment and management (means scores below 5/10).

#### Attitudes

**Fig. 2:**
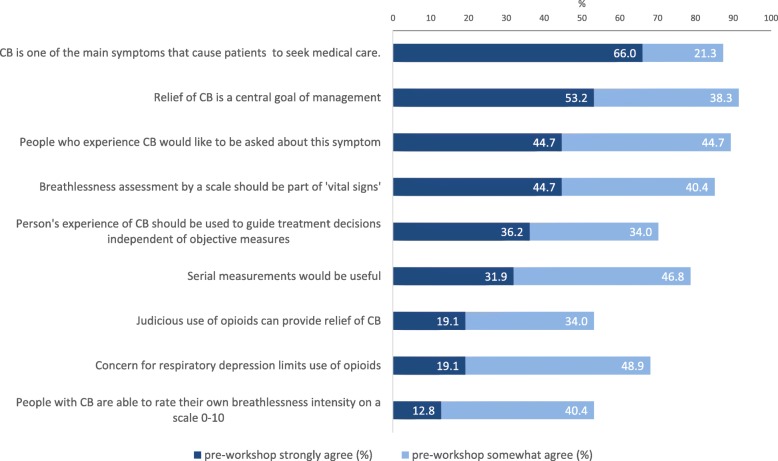
Baseline attitudes of participants (*n* = 47) regarding chronic breathlessness assessment and management. Bars indicate percentage of participants who agreed with statements (selected strongly agree and somewhat agree from 5-point Likert-style responses; items modified from Stefan et al. [20] with permission). CB = chronic breathlessness

Baseline responses (proportion of *n* = 47 participants,who ‘strongly agree’ and ‘somewhat agree’ with Likert statements) are presented in Fig. 2. Internal consistency of the these nine Likert-style items was acceptable (Cronbach’s alpha = 0.893). There were two items for which the majority of respondents strongly agreed at baseline: chronic breathlessness was a major symptom causing people with advanced cardiopulmonary conditions and cancer to seek care (66%), and relief from this symptom was a central goal of management (53.2%). Under half of respondents strongly agreed that people who experience chronic breathlessness would like to be asked about this symptom (44.7%) or that a person’s experience of breathlessness independent of objective measures should be used to guide treatment decisions (36.2%). Less than 13% of respondents strongly agreed that people with chronic breathlessness are able to rate their own breathlessness intensity on a 0–10 scale.

Neutral responses were highest for the statement that judicious use of opioids can provide relief of chronic breathlessness (36% neutral) and that people are able to rate their own breathlessness intensity on a scale (21% neutral). Fewer than 9% of participants strongly disagreed with any statement. Nineteen percent somewhat disagreed that people with chronic breathlessness are able to rate their own breathlessness intensity on a scale, but for all other statements, the proportion who somewhat disagreed was less than 7%. Two or three participants left every response blank in this section.

Baseline responses to non-Likert style questions on current practice in breathlessness assessment and management are presented in Table 4. Two thirds of respondents assessed chronic breathlessness at a client’s first consultation, around half at final consultation, and 38.3% at each consultation. The majority (55.3%) asked the client to rate their own severity of breathlessness using a categorical scale. Most respondents indicated that awareness of breathlessness severity impacted their management of patients by adding non-pharmacological symptom-oriented strategies (83%), reviewing existing strategies including inhaler use (72.3%) or referring to additional services (68%). Fewer described that awareness of breathlessness severity influenced pursuit of additional diagnostic testing (34%) or changed decision-making around timing of discharge for hospitalised patients (23%, Table 4). Non-pharmacological therapies thought to be effective by more than 85% of respondents were pursed lip breathing, pulmonary rehabilitation/exercise training, pacing/fatigue management, positioning to alleviate breathlessness walking aids and home modification, cool air/fan and relaxation techniques (Table 4). Notably 14 (29.8%) respondents indicated that supplemental oxygen for non-hypoxaemic patients was an effective strategy for relief of chronic breathlessness.

**Table 4:** Baseline responses regarding current practices in assessment and management of chronic breathlessness (non-Likert style questions, *n* = 47)

Question and response options	frequency(%)
**When caring for people with chronic cardiopulmonary disease/cancer how often do you assess severity of breathlessness?** (select as many as apply)	
At admission/initial consultation	31 (66.0)
At discharge/final consultation	24 (51.1)
Daily until discharge/each occasion of service	18 (38.3)
With all outpatient/ambulatory reviews	18 (38.3)
More often than daily/more often than once each occasion of service	9 (19.1)
**Which description best characterises your approach to assessing breathlessness severity?**	
I ask the patient to rate the severity of shortness of breath using a categorical scale (e.g. somewhat SOB, no SOB, improved or worsened compared with a prior date)	26 (55.3)
Other^a^	9 (19.1)
I ask the patient whether or not they are having shortness of breath	7 (14.9)
Blank	3 (6.4)
I don’t regularly ask about breathlessness severity	2 (4.3)
**Awareness of breathlessness severity affects my management by influencing my decision:** (select all that apply)	
To add non-pharmacologic-based, symptom-oriented treatment for breathlessness, such as fans or pursed lip breathing technique	39 (83.0)
To review current strategies to manage breathlessness including inhaler use	34 (72.3)
To refer person on for additional therapeutic or social servicesIncluding: palliative care/psychology/other	32 (68.1)
To intensify treatment of the patient’s underlying condition	24 (51.1)
To add/refer for pharmacologic-based, symptom-oriented treatment for breathlessness, such as opioids	19 (40.4)
To pursue additional diagnostic testing	16 (34.0)
Regarding timing of discharge (for hospitalised people)	11 (23.4)
**Which of the following non-pharmacological/non-surgical therapies are effective for the relief of chronic breathlessness?** (select all that apply)	
Pursed lip breathing	43 (91.5)
Pulmonary rehabilitation/exercise training	42 (89.4)
Pacing/fatigue management	42 (89.4)
Positioning to alleviate breathlessness	41 (87.2)
Relaxation techniques	40 (85.1)
Cool air/fan	40 (85.1)
Walking aids and home modification	40 (85.1)
Mindfulness techniques	37 (78.7)
Cognitive behavioural strategies	34 (72.3)
Non-invasive ventilation	23 (48.9)
Oxygen for non-hypoxaemic patients	14 (29.8)
Other (free text responses:don’t know; high flow nasal cannula; some patients report use of airway clearance devices help their breathlessness	3 (6.4)

^a^Free text associated with “other”indicated use of the Borg scale, asking whether or not they are having shortness of breath and observation at rest and during functional assessment; Symptom Assessment Scale for distress about SOB; just ask them to describe it to me

*SOB* shortness of breath

### Pre-post workshop change: immediate evaluation

#### Changes in in familiarity and confidence

**Fig. 3:**
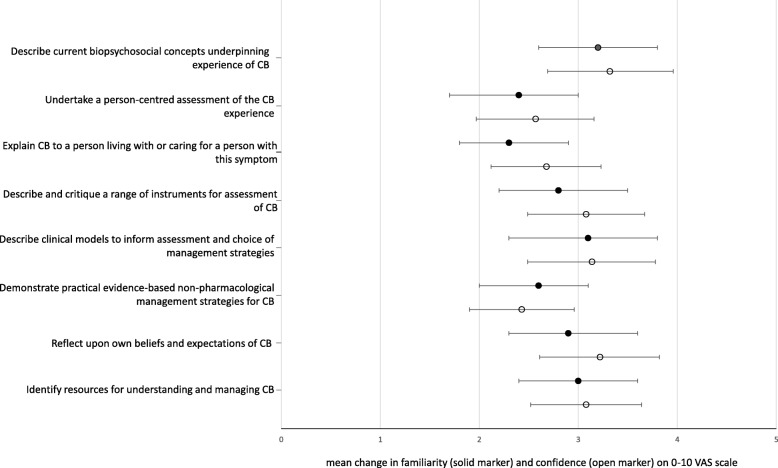
Pre-post workshop changes in participant self-ratings of familiarity (*n* = 39) and confidence (*n* = 37) with workshop objectives. Markers indicates mean pre-post workshop change in self-rated familiarity (solid markers) on visual analogue scale (VAS) where 0 = very unfamiliar, 10 = very familiar; and self- confidence self-rated confidence (open markers) on VAS where 0 = not at all confident, 10 = very confident, for each workshop objective. Error bars represent 95% confidence interval (CI) of change. CB = chronic breathlessness

Thirty nine participants submitted both pre and post workshop surveys, thus changes in knowledge, skills, and attitudes are reported based on available paired data (two respondents completed familiarity measures but left all other answers blank). Compared to pre-workshop ratings, familiarity with all eight knowledge and skills items was significantly greater post-workshop (*n* = 39, Fig. 3). The greatest gains were demonstrated in familiarity with the biopsychosocial concepts underpinning the experience of chronic breathlessness (mean change = 3.2 points; 95%CI 2.6 to 3.8 on VAS 0–10 scale, *p* < 0.001, effect size/Cohen’s d = 1.9), clinical models to inform assessment, and choice of management strategies (3.1; 2.3 to 3.8, *p* < 0.001, d = 1.5), and identifying resources for understanding and managing chronic breathlessness (3.0; 2.4 to 3.6, *p* < 0.001, d = 1.8).

Similarly, confidence to demonstrate all course objectives significantly improved (*n* = 37, Fig. 3) with the greatest improvements shown in confidence to describe current biopsychosocial concepts (3.2, 2.7 to 4.0, *p* < 0.001, d = 1.9), reflect upon own beliefs and expectations (3.2, 2.6 to 3.8, *p* < 0.001, d = 1.9), and describe clinical models (3.1, 2.5 to 3.8, *p* > 0.001, d = 1.5).

#### Changes in attitudes

Pre and post workshop change in attitudes about breathlessness assessment and management in matched pair responses (*n* = 37) are displayed in Table 5 . The largest shift in attitude was toward agreement that a person’s experience of breathlessness should be used to guide treatment decisions, independent of objective measures such as respiratory rate and oxygen saturation (effect size, *r* = 0.6). Post-workshop, 100% of participants agreed with this statement (73% strongly) compared with 73% pre-workshop (43% strongly). Respondents significantly changed their belief toward agreement that people are able to rate their own degree of chronic breathlessness on a scale (effect size, *r* = 0.5); however only a minority strongly agreed with this statement at both time-points (pre-workshop 11%, post-workshop 22%). Post-workshop there was a significant change toward agreement that the person with breathlessness would like to be asked about this symptom (effect size, *r* = 0.4; from 43 to 76% in strong agreement).

**Table 5:** Pre-post course change in attitudes, Likert-style responses (n = 37 matched pairs)

Statement	Pre-workshop *n* = 37		Post-workshop *n* = 37		Pre-post change
	strongly agree %	somewhat agree %	strongly agree %	somewhat agree %	Wilcoxon signed rank test z statistic, *p* value, effect size
Person’s experience of CB should be used to guide treatment decisions independent of objective measures	43.2	29.7	73.0	27.0	**3.74, < 0.001, 0.6**
People with CB are able to rate their own breathlessness intensity on a scale 0–10	10.8	48.6	21.6	59.5	**2.95, 0.003, 0.5**
People who experience CB would like to be asked about this symptom	45.9	48.6	73.0	27.0	**2.68, 0.007, 0.4**
Serial measurements would be useful	35.1	45.9	43.2	56.8	1.98, 0.05, 0.3
Breathlessness assessment by a scale should be part of ‘vital signs’	45.9	43.2	62.2	35.1	1.97, 0.05, 0.3
Judicious use of opioids can provide relief of CB	21.6	37.8	27.0	54.1	1.65, 0.1, 0.3
Concern for respiratory depression limits use of opioids	21.6	51.4	21.6	64.9	0.96, 0.34, 0.2
Relief of CB is a central goal of management	51.4	43.2	64.9	27.0	0.89, 0.37, 0.1
CB is one of the main symptoms that cause patients to seek medical care.	67.6	24.3	67.6	24.3	0.0, 1, 0

*CB* chronic breathlessness; Bold text indicates significant pre-post change in Wilcoxon signed rank test, *p* < 0.05

Reponses to non-Likert type questions (Additional File 3) showed that after the workshop, more participants nominated “cognitive behavioural therapy” as an effective therapy for the relief of chronic breathlessness (81% versus 100%, significantly more likely to change in a positive direction, McNemars test statistic = 5.14, *p* = 0.016). More participants also changed their view (disagree to agree) that assessment of breathlessness intensity would influence their decision regarding timing of discharge (26% vs 46%, test statistic = 4.08, *p* = 0.04). While fewer participants supported the use of oxygen for non-hypoxaemic patients post-workshop (32% pre versus 14% post), the proportion of respondents changing their view (agree to disagree) was not significant (test statistic = 3.273, *p* = 0.07).

#### Partcipant plans for implementation of information and skills

Post-workshop respondents (*n* = 41) indicated they planned to use the course information and skills in clinical practice (85% of respondents), teaching other health professionals (71%) or teaching students (49%), and in service design (45%), policy/protocol development (10%) and research (24%).

Free text reponses (Additional File 4) included multiple (*n* = 13) specific statements about immediate or planned impact of the course in changing participants’ clinical practice; integrating new knowledge into clinical practice; introducing new concepts; re-framing thinking about chronic breathlessness; sharing new information with their clinical team and recommending the course to other health professionals.

The main barriers anticipated to implementation (Additional File 5) were workplace culture, service models, and organisational resources (*n* = 10 comments) along with lack of time and prioritisation to facilitate practice of assessment and intervention strategies (10). Accessing materials such as fans and teaching resources (*n* = 7) and a need for further knowledge and skills (6), either as individuals or for their healthcare team, were also identified as barriers. A number of participants documented a planned way to overcome potential barriers and one respondent specifically suggested they anticipated no barriers.

#### Workshop feedback: Most helpful/least helpful aspects and suggested changes

Participant responses indicated that the workshop was positively received, facilitated learning through its structure, delivery and organisation, and stimulated discussion and collaboration (Additional File 4). A total of 87 participant comments related to perceived “most helpful” aspects and 20 to “least helpful” aspects of the workshop. Conversation sessions about breathlessness with people living with this symptom featured in both categories (most helpful, 13; least helpful, 9). Other highly valued aspects were the workshop resources (10 comments), content on psychological aspects of breathlessness and the “thinking” part of the BTF model (10), and the fan and associated “breathing” strategies (7). Suggested changes included opportunities to observe assessments and interventions in action (3 comments) and online course delivery (3).

## Discussion

In this group of nursing and allied health clinicians experienced in the care of people with chronic breathlessness, baseline familiarity and confidence with multidimensional assessment, biopsychosocial understanding and explaining chronic breathlessness was moderate on average. Strong agreement at baseline about the importance of chronic breathlessness in clinical management contrasted with much less strong agreement about how to operationalise this in assessment or treatment decision making. The immediate impact of this three-day training workshop, centered around a biopsychosocial understanding of chronic breathlessness, was to improve familiarity and confidence in all areas, change key attitudes and facilitate professionals’ plans to implement changes in their practice.

### Gaps in baseline confidence, familiarity with and attitudes about chronic breathlessness

Over the past two decades, changes in understanding the neurophysiological as well as pathophysiological components of chronic breathlessness have prompted new strategies for assessment [1, 28], clinical interventions and service delivery [6]. Similarities between the trajectories of basic and implementation science for chronic pain and chronic breathlenessness are well recognised [29, 30], with breathlessness science lagging several decades behind [8]. The application of the biopsychosocial model of pain perception has been revolutionary in driving a re-conceptualisation of pain [31] and in using neuroscience education to change attitudes, physical and psychological performance and reduce disability in people experiencing chronic pain [32–35].

In contrast, there is relatively little is known about clinician attitudes, understanding and confidence in chronic breathlessness assessment and management. Recent surveys of medical professionals concerning dyspnoea reflect positive perceptions of the value of standardised assessments in patient care (*n* = 255 hospital doctors [23]), but less consistent agreement about the ability of patients to rate their dyspnoea on a scale (42% [26]). A survey of respiratory and palliative care doctors in Australia and New Zealand also found that a minority reported using a breathlessness scale in clinical practice (30 and 18% for respiratory and palliative care respectively) [22].

The current evaluation is the first to examine a similar question in allied health and nursing professionals, with a somewhat higher proportion agreeing that people with chronic breathlessness were able to self-rate severity of the symptom (53% pre-workshop), or indicating that they ask clients to rate their breathlessness (55%). In comparison with previous reports this may indicate a shift in attitude over time, or a difference between medical and non-medical health professionals. It still represents less than ideal agreement, given existing recommendations by peak respiratory professional associations that breathlessness should be rated by the person experiencing it and used to guide treatment decisions, in the same way as pain ratings [1, 36]. Seeking, valuing and acting on the perceptions of people with breathlessness is essential to providing person-centred clinical care and part of wider partnerships with consumers in the planning, design, delivery, measurement and evaluation of care, an evidence-based national standard for safety and quality in health care [37].

Our findings of moderate baseline familiarity and confidence and mixed attitudes about assessment and management are not surprising, given under-recognition of chronic breathlessness that persists despite optimal treatment of the underlying disease conditions [2]. Interdisciplinary workforce training about chronic breathlessness management potentially offers a way to address this issue [38]. International curricula and competencies for palliative care training are established [39, 40], with breathlessness as one of many topics that they address. Education initiatives for health professional training in palliative care have existed in Australia since at least 2003 [41, 42] accompanied by free online resources [43]. Some degree of implementation in health undergraduate education has also occurred, including topics of symptom management and non-pharmacological interventions [44, 45]. However, a recent systematic review indicated that general medical specialist training in end-of-life care symptom management focused on pain management using opioids [46], with less emphasis on preparedness to manage other symptoms including breathlessness. Beyond palliative care training, integration of contemporary chronic breathlessness understandings and management earlier in the care trajectory of chronic conditions is recommended [5, 47]. Accordingly, a recent European post-graduate respiratory physiotherapy curriculum includes dyspnoea-related knowledge, skills and attitudes [48] and inclusion of chronic breathlessness on the agendas of all such education initiatives is an important step.

### Attitude, familiarity and confidence change through challenging and re-forming assumptions

Rather than a focus on didactic skills teaching, this workshop sought to promote effective conceptual change [49] using a transformational learning framework [25]. According to this theory, learners radically change their existing beliefs and understandings through reflection on how they were originally learned and practical experience; exposure to and empathy with alternative viewpoints; discourse in a supportive environment between old and new understandings, resulting in a planned course of action [50, 51]. This is in common with conceptual change learning in pain neurophysiology education [31] and has resonance with the “challenging misconceptions” concept of the BTF clinical model itself [14]. For example, to promote learning in relation to the issue of breathlessness assessment and use in directing therapy, we first used reflection and description to activate participants’ prior understandings. Conversations about breathlessness directed by the consumers themselves, as well as insights into research on the neurophysiology of breathlessness, highlighted the nature and implications of the person’s experience. Input from a pain science researcher prompted participants to make links between new understandings in chronic breathlessness and chronic pain. Interactive sessions then gave exposure to and practice using current multi-dimensional breathlessness assessment tools, in readiness for planned implementation in clinical settings.

Shifts in three important attitudes provide evidence of the effectiveness of this approach in the immediate post-workshop period. Firstly, more participants agreed that people with chronic breathlessness would like to be asked about this symptom. Secondly, more participants believed that a person’s experience of chronic breathlessness should be used to guide treatment decisions: this was the largest change in attitude observed, from 43 to 73% strong agreement post-workshop. Thirdly, a significant change occurred in the belief that people with chronic breathlessness could rate the intensity of this symptom on a numeric scale took place, although at 81% with any level of agreement post-workshop (only 22% strong) this still remained a disputed concept. Given that this concept had the lowest agreement pre-workshop, and was similarly low in previous cross-sectional studies [22, 26], further exploration is warranted to explain reasons why this belief is retained by many experienced clinicians.

Improvements demonstrated in familiarity and confidence in all learning objectives of this workshop builds on previous reports that focused primarily on skills development [19, 20]. In our study, greatest changes in confidence and familiarity were observed in the biopsychosocial concepts underpinning experience of chronic breathlessness; the BTF clinical model and with participants’ ability to reflect on their own beliefs and expectations of chronic breathlessness and how these may contribute to the client experience and management. These are the kinds of paradigm changes in understanding and expectations that are likely to influence clinician behaviour in a longer lasting way than skill development alone.

#### Implications for clinical practice

Meaningful changes in health care professional awareness, understanding and thus ability to respond to people’s experience, needs, and preferences regarding breathlessness are vital components of enabling people with chronic breathlessness to attain better quality of life. Using systematic review and meta-synthesis of 101 qualitative studies, Hutchinson and colleagues [52] described three major themes that interact to promote best possible participation in life for people living with breathlessness. Responsive clinical interactions with health professionals was one of these themes, combined with engaged coping and help-seeking on the part of the person with breathlessness. Important elements of clinician responsiveness to breathlessness identified included the person’s experience being taken into account, forming a shared understanding of the impact of breathlessness; and avoiding a clinician response that suggests “nothing more can be done” [52]. Early post-workshop changes in attitudes, familiarity, and confidence after this training directly reflected each of these important aspects. Participants were better equipped to seek out and value and the person’s experience of chronic breathlessness in the assessment and clinical decision making process. Improved familiarity with bio-psychosocial understandings, the BTF clinical model and associated resources prepared participants to offer non-pharmacological management options including a changed attitude supporting cognitive behavioural strategies. Post-workshop, participants indicated their intent to change clinical practice and share their learnings with other health professionals.

A limitation of this study was that the underlying structure of the questionnaire items used to assess familiarity, confidence and attitudes had not previously been reported. We conducted exploratory factor analysis using baseline data questionnaire responses for each construct (Additional File 6), identifying a single construct underlying each of familiarity and confidence, and a three-factor structure underlying the nine Likert-style attitude items. While factor scores for familiarity and confidence were strongly correlated (*r* = 0.965, *p* < 0.001, Additional File 6), these were not related to any of the three attitude factors. This suggests that at baseline, familiarity and confidence with the course objectives were closely related to each other, but attitudes measured by the questionnaire items were not related at baseline to these aspects of knowledge or skill. A wide range of other factors not encapsulated by the familiarity/confidence items may influence clinician attitudes regarding chronic breathlessness. This is consistent with research using functional neuroimaging showing how a person’s prior experience and emotions contribute to the brain networks that shape perception of breathlessness through inference from predictions [53]. Previous clinician experiences and expectations may similarly shape attitudes such as hope and optimism versus helplessness [54]. Our survey questions did not include concepts such as breathlessness catastrophizing that may shed light on clinician behaviour and could be included in a future evaluation. Further psychometric development of measures to represent and evaluate clinician attitudes toward the assessment and management of chronic breathlessness is indicated.

Findings of this study are limited to participants’ self-reported changes in familiarity, confidence, attitude and planned behaviour in the immediate post-workshop period. Reporting of participant follow-up at 6 months after workshop completion is planned, including documentation of examples of changed clinician practices. Future research should examine outcomes for people with chronic breathlessness (eg. changes in breathlessness unpleasantness ratings, quality of life, anxiety) as a result of changed clinician behaviour.

## Conclusion

This evaluation of the immediate effects of a health professional training workshop demonstrated pivotal changes in participant attitudes, understandings and confidence in the non-pharmacological management of chronic breathlessness. The unique focus on changed biopsychosocial understandings of chronic breathlessness and involvement of people living with this symptom were valued and identified as promoters of this change. Future reports will examine the retention of these changed beliefs and understandings and the impact of the training over the longer term.

## Supplementary information

**Additional file 1.** Detail of 3-day workshop Practical Management of Chronic Breathlessness

**Additional file 2.** Survey questions for evaluation of health professional training workshop

**Additional file 3.** Pre-post workshop change in current practice in assessment and management of chronic breathlessness: non-Likert response questions

**Additional file 4.** Summarised free-text responses to post-workshop open questions about most and least helpful aspects, suggested changes and any other comments.

**Additional file 5.** Potential barriers to implementation anticipated by the post-course respondents

**Additional file 6.** Exploratory factor analysis of items for familiarity, confidence and attitudes.

## Data Availability

All data generated or analysed during this study are included in this published article and its supplementary information files.

## Abbreviations

BTF: Breathing, Thinking, Functioning
CI: Confidence interval
IQR: Interquartile range
SD: Standard deviation
VAS: Visual analogue scale
