# Supplementary material for: Attitude change and increased confidence with management of chronic breathlessness following a health professional training workshop: a survey evaluation
Source: BMC Med Educ. 2020 Mar 30;20:90. doi: 10.1186/s12909-020-02006-7 (PMC7106669; doi:10.1186/s12909-020-02006-7)
Supplement: Supplementary file 1 — Additional file 1. Detail of 3-day workshop Practical Management of Chronic Breathlessness [file 12909_2020_2006_MOESM1_ESM.docx]

**Additional File 1**: Detail of 3-day workshop Practical Management of Chronic Breathlessness

| DAY 1: Assessment and the Breathing vicious cycle | | | | |
| --- | --- | --- | --- | --- |
| **Session title** | **Duration** | **Instructor(s)** | **Teaching and learning delivery and strategy** | **Materials** |
| Welcome, introductions and overview | 15min | KJ | informal presentation |  |
| The genesis of breathlessness: models and mechanisms | 30min | MTW | presentation | workbook |
| Introduction to the Breathing, Thinking, Functioning model | 30min | SB | presentation | workbook |
| Understanding breathlessness: What is really like to live with? | 45min | KJ, consumer volunteer A | interview/ conversation; interactive questions and answers |  |
| Assessing breathlessness | 60min | MTW | interactive practical session with small group activities | workbook activities |
| Conversations about living with breathlessness | 45min | SB, consumer volunteer B | interview conversation; interactive questions and answers |  |
| Modifying the breathing vicious cycle | 60min | KJ, SB | combined presentation with interactive practical components | workbook, toolkit, fan |
| Summary and reflection | 60 mins | MTW | facilitated reflection activities; *things learned*, interactive *questions* and answers |  |
| DAY 2: Thinking vicious cycle: beliefs and expectations | | | | |
| **Session title** | **Duration** | **Instructor(s)** | **Teaching and learning delivery and strategy** | **Materials** |
| Overview of the day | 15 mins | MTW | informal presentation |  |
| Conversations about living with breathlessness | 30 mins | SB, consumer volunteer C | interview conversation; interactive questions and answers |  |
| Introduction to the Thinking domain | 30 mins | SB | presentation | workbook |
| What does Thinking include and how do we explain breathlessness? | 45 mins | MTW | presentation | workbook |
| What lessons can we learn from pain neuroscience education? | 60 mins | guest speaker | presentation | workbook |
| Modifying the Thinking vicious cycle: practical strategies | 60 mins | guest speaker | combined presentation with interactive practical components; small group case study activity with feedback | workbook activities |
| Modifying the Thinking vicious cycle: explaining breathlessness | 60 mins | MTW | interactive practical session with small group activities; *group responses collected* | workbook activities |
| Summary and reflection | 30 mins | SB | facilitated reflection activities; *things learned*, interactive questions and answers |  |

| DAY 3: Functioning vicious cycle | | | | |
| --- | --- | --- | --- | --- |
| **Session title** | **Duration** | **Instructor(s)** | **Teaching and learning delivery and strategy** | **Materials** |
| Overview of the day | 15 mins | SB | informal presentation |  |
| The Functioning domain: understanding and management | 60mins | KJ , MY | Presentation, including brief informal contributions from course participants | workbook, toolkit, pedometer |
| Conversations about living with breathlessness: carer perspective | 30mins | SB, DK | interview conversation; interactive questions and answers |  |
| Informal carers for people living with breathlessness | 15mins | KJ | presentation | workbook |
| Pharmacological management / crisis management | 30mins | SB | presentation | workbook |
| Integration of the palliative approach into models of care in chronic respiratory conditions | 30mins | MY | presentation | workbook |
| Integrating all aspects of the BTF clinical model | 60mins | SB | interactive small group case study activity with feedback | workbook activities |
| Service models: national and international | 60 mins | SB | facilitated discussion including brief informal contributions from course participants | workbook resources |
| Resources for clinicians and people with breathlessness |  |  | listing of all references and links to online resources including video, audio | workbook resources |
| Summary and reflection | 30 mins | MTW | *facilitated reflection activities* |  |

*italics indicates data collected in session*

**Instructor professional discipline and experience**

SB: palliative care physician, 30+ years’ experience and publications in research and clinical services in breathlessness; internationally recognised expert in the field.

MTW: physiotherapist/academic with 25+ years’ experience in cardiorespiratory physiotherapy, research conduct and training; 15 years’ experience and publications in research in breathlessness assessment, clinical services

KJ: physiotherapist/academic with 25+ years’ experience in cardiorespiratory physiotherapy; 10 years’ experience university teaching and research; 5 years experience and publications in breathlessness related research.

MY: respiratory nurse practitioner with 30+ years clinical, management and policy experience; 12 years’ experience translational research and publications.

DK: consumer, carer and consumer representative with 30+ years’ experience and national leadership in training, policy and advocacy; multiple associated national board and committee appointments.

**Workshop delivery**

Daily introductions and summaries provided structured space for self-reflection, exchange of questions and answers and participant goal setting.

Small group practice in the focused application of workshop content to case study examples and sharing of clinical experience in the delivery of services to people with chronic breathlessness were included. This promoted active exchange of information between participants and planning for translation of workshop content into clinical practice.

Regular breaks with in-room catering facilitated opportunities for participant-directed conversations with each other and with course facilitators.

Participants received a daily workbook with printed session content (slides, course activities, assessment tools, references and resources) and a ‘Breathing Repair Toolkit’ of memo cards related to intervention strategies (used with kind permission of the Cambridge Breathlessness Intervention Service), a handheld fan and a pedometer.
