# Supplementary material for: Attitude change and increased confidence with management of chronic breathlessness following a health professional training workshop: a survey evaluation
Source: BMC Med Educ. 2020 Mar 30;20:90. doi: 10.1186/s12909-020-02006-7 (PMC7106669; doi:10.1186/s12909-020-02006-7)
Supplement: Supplementary file 2 — Additional file 2. Survey questions for evaluation of health professional training workshop [file 12909_2020_2006_MOESM2_ESM.docx]

**Additional File 2**: Survey questions for evaluation of health professional training workshop.

| Section 1: Information about you: Demographics (Pre-workshop survey only) 8 questions | |
| --- | --- |
| Male/female | m/f |
| Age (in years) | <free text> |
| Profession | <free text> |
| Professional role in the last 6 months | Mainly clinical  (i.e. direct patient management)  Mainly non-clinical  (e.g. academic /research/administrative) |
| If mainly clinical: What is the main setting for your clinical practice? | Hospital (public)  Hospital (private)  Community based: private practice  Community based: non-government organisation  Community based: palliative care  Community based: aged care  Primary/intermediate care  Residential aged care facility  other |
| If mainly non-clinical: What is the main setting for your work? | University: teaching  University: research  Hospital: management  Hospital: research  Other |
| Years of practice (with people with chronic breathlessness) | <free text> |
| Frequency of conversation about chronic breathlessness in the past 3 months | At least once a day-  At least once a week-  At least once a month-  Not at all  No direct clinical contact with people with chronic breathlessness |
| Expertise in chronic breathlessness | sliding numerical rating scale (NRS)  Self-rated where 0 = ‘No understanding of theoretical and/or clinical aspects’ and 100 = ‘Extensive understanding of theoretical and/or clinical aspects’) |

| Section 2: Experience in aspects of practical management of chronic breathlessness (all surveys). Modified from Froggatt et al 2005 | |
| --- | --- |
| Please rate your level of familiarity now to: |  |
| Describe current biopsychosocial concepts underpinning the experience of chronic breathlessness | NRS scale 0 to 10  0= very unfamiliar, 10=very familiar |
| Undertake a person-centred assessment of the breathlessness experience and symptom needs of a person living with or caring for a person living with this symptom | NRS scale 0 to 10  0= very unfamiliar, 10=very familiar |
| Explain chronic breathlessness to a person living with or caring for a person living this symptom using jargon and value - free language | NRS scale 0 to 10  0= very unfamiliar, 10=very familiar |
| Describe and critique a range of instruments appropriate for assessment and monitoring chronic breathlessness. | NRS scale 0 to 10  0= very unfamiliar, 10=very familiar |
| Describe clinical models to inform assessment and choice of management strategies | NRS scale 0 to 10  0= very unfamiliar, 10=very familiar |
| Demonstrate practical, evidence-based non-pharmacological management strategies for chronic breathlessness. | NRS scale 0 to 10  0= very unfamiliar, 10=very familiar |
| Reflect upon own beliefs and expectations of chronic breathlessness and how these may contribute to the client experience and management. | NRS scale 0 to 10  0= very unfamiliar, 10=very familiar |
| Identify resources for understanding and managing chronic breathlessness. | NRS scale 0 to 10  0= very unfamiliar, 10=very familiar |

| Section 3: Confidence in aspects of practical management of chronic breathlessness (all surveys)  Modified from Froggatt et al 2005 | |
| --- | --- |
| Please rate how confident you feel to: |  |
| Describe current biopsychosocial concepts underpinning the experience of chronic breathlessness | NRS scale 0 to 10  0= not at all confident, 10=very confident |
| Undertake a person-centred assessment of the breathlessness experience and symptom needs of a person living with or caring for a person living with this symptom | NRS scale 0 to 10  0= not at all confident, 10=very confident |
| Explain chronic breathlessness to a person living with or caring for a person living this symptom using jargon and value - free language | NRS scale 0 to 10  0= not at all confident, 10=very confident |
| Describe, critique and apply a range of instruments appropriate for assessment and monitoring chronic breathlessness. | NRS scale 0 to 10  0= not at all confident, 10=very confident |
| Describe and apply clinical models to inform assessment and choice of management strategies | NRS scale 0 to 10  0= not at all confident, 10=very confident |
| Demonstrate practical, evidence-based non-pharmacological management strategies for chronic breathlessness. | NRS scale 0 to 10  0= not at all confident, 10=very confident |
| Reflect upon own beliefs and expectations of chronic breathlessness and how these may contribute to the client experience and management. | NRS scale 0 to 10  0= not at all confident, 10=very confident |
| Identify resources for understanding and managing chronic breathlessness. | NRS scale 0 to 10  0= not at all confident, 10=very confident |

(Pre-workshop only): Are there additional topics/issues you would like to see addressed in this workshop? Please list them: <free text>

Section 4: Attitudes toward breathlessness assessment and management (all surveys) (modify from Stefan et al 2015)

| 1 | Chronic breathlessness is one of the main symptoms that cause patients with advanced cardiopulmonary conditions (e.g. COPD, heart failure, interstitial lung disease) and cancer to seek medical care. | - Strongly disagree - Somewhat disagree - Neutral - Somewhat agree - Strongly agree |
| --- | --- | --- |
| 2 | Relief of breathlessness is a central goal of the management of patients with advanced cardiopulmonary conditions/cancer. | - Strongly disagree - Somewhat disagree - Neutral - Somewhat agree - Strongly agree |
| 3. | When caring for people with chronic cardiopulmonary diseases/cancer how often do you assess severity of breathlessness? | Select all that apply   - At admission/initial consultation - At discharge/final consultation - Daily until discharge/each occasion of service - More often than daily/more often than once each occasion of service - With all outpatient/ambulatory reviews - Other: Free text for <other> |
| 4. | Which description best characterizes your approach to assessing breathlessness severity? | Select one   - I don't regularly ask about breathlessness severity - I ask the patient whether or not they are having shortness of breath - I ask the patient to rate the severity of shortness of breath using a numeric scale - I ask the patient to rate the severity of shortness of breath using a categorical scale (e.g. somewhat SOB, no SOB, improved or worsened compared with a prior date) - Other: Free text for “other” |
| 5. | Awareness of breathlessness severity affects my management by: | (Select all that apply)   - Influencing my decision to intensify treatment of the patient's underlying condition - Influencing my decision to pursue additional diagnostic testing - Influencing my decision to review current strategies to manage breathlessness including inhaler use - Influencing my decision to add/refer for pharmacologic-based, symptom-oriented treatment for breathlessness, such as opioids - Influencing my decision to add non-pharmacologic-based, symptom-oriented treatment for breathlessness, such as fans or pursed lip breathing technique - Influencing my decision regarding timing of discharge (for hospitalised people) - Influencing my decision to refer person on for additional therapeutic or social services   Including: palliative care/psychology/other  Add free text space for “other” |
| 6. | People who experience chronic breathlessness would like me to ask them about this symptom. | - Strongly disagree - Somewhat disagree - Neutral - Somewhat agree - Strongly agree |
| 7. | People who experience chronic breathlessness are able to rate their own breathlessness intensity on a scale of 0-10. | - Strongly disagree - Somewhat disagree - Neutral - Somewhat agree - Strongly agree |
| 8. | Serial measurements of breathlessness would be useful for assessing response to therapy. | - Strongly disagree - Somewhat disagree - Neutral - Somewhat agree - Strongly agree |
| 9. | Breathlessness assessment by a scale should be part of the "vital signs" for patients with cardiopulmonary diseases. | - Strongly disagree - Somewhat disagree - Neutral - Somewhat agree - Strongly agree |
| 10. | The person’s experience of chronic breathlessness should be used to guide treatment decisions independent of objective measures such as respiratory rate and oxygen saturation. | - Strongly disagree - Somewhat disagree - Neutral - Somewhat agree - Strongly agree |
| 11. | Judicious use of oral and/or parenteral opioids can provide relief of chronic breathlessness. | - Strongly disagree - Somewhat disagree - Neutral - Somewhat agree - Strongly agree |
| 12. | Limited use of opioids for relief of chronic breathlessness in patients with advanced cardiopulmonary disorders is often due to concerns of respiratory depression. | - Strongly disagree - Somewhat disagree - Neutral - Somewhat agree - Strongly agree |
| 13. | Which of the following non-pharmacologic/non-surgical therapies are effective for the relief of chronic breathlessness? | (Select all that apply)   - Pursed lip breathing - Relaxation techniques - Mindfulness techniques - Non-invasive ventilation - O2 for non-hypoxemic patients - Cool air/fan - Cognitive behavioural strategies - Pulmonary rehabilitation/Exercise training - Pacing and fatigue management - walking aids and home modifications - positioning to alleviate breathlessness - other: <Plus free text for other> |

Section 5: Workshop feedback and impact planning (post workshop 1 week)

| What aspects, features or content of this workshop did you find **most** helpful? | Free text |
| --- | --- |
| What aspects, features or content of this workshop did you find **least** helpful? | Free text |
| Can you suggest any changes to the workshop? | Free text |
| How do plan to use information and skills gained from this workshop? | Select all that apply   - In clinical practice - In teaching to other health professionals? - In teaching to students? - In service design? - In protocol or policy development - In research - In any other aspects of your work? <free text> |
| Please describe any barriers you anticipate to implementing information and skills gained from this workshop in your work | Free text |

Section 6: Any other comments (all surveys)

| Are there any other comments you would like to make? | Free text |
| --- | --- |
