# Supplementary material for: Attitude change and increased confidence with management of chronic breathlessness following a health professional training workshop: a survey evaluation
Source: BMC Med Educ. 2020 Mar 30;20:90. doi: 10.1186/s12909-020-02006-7 (PMC7106669; doi:10.1186/s12909-020-02006-7)
Supplement: Supplementary file 3 — Additional file 3. Pre-post workshop change in current practice in assessment and management of chronic breathlessness: non-Likert response questions [file 12909_2020_2006_MOESM3_ESM.docx]

**Additional File 3**: Pre-post workshop change in current practice in assessment and management of chronic breathlessness: non-Likert response questions (n=37, paired data)

|  | **Pre-workshop**  frequency (%), n=37 | **Post-workshop**  frequency (%), n=37 |
| --- | --- | --- |
| **When caring for people with chronic cardiopulmonary disease/cancer how often do you assess severity of breathlessness?** (select as many as apply) | | |
| At admission/initial consultation | 24(64.9) | 25(67.6) |
| At discharge/final consultation | 18(48.6) | 17(45.9) |
| Daily until discharge/each occasion of service | 15(40.5) | 15(40.5) |
| With all outpatient/ambulatory reviews | 15(40.5) | 24(64.9) |
| More often than daily/more often than once each occasion of service | 8(21.6) | 4(10.8) |
|  |  |  |
| **Which description best characterises your approach to assessing breathlessness severity?** | | |
| I ask the patient to rate the severity of shortness of breath using a categorical scale (e.g. somewhat SOB, no SOB, improved or worsened compared with a prior date) | 20(54.1) | 26(70.3) |
| Other* | 8(21.6) | 5(13.5) |
| I ask the patient whether or not they are having shortness of breath | 7(18.9) | 5(13.5) |
| I don’t regularly ask about breathlessness severity | 2(5.4) | 1(2.7) |
|  | | |
| **Awareness of breathlessness severity affects my management by (select all that apply)** | | |
| Influencing my decision to add non-pharmacologic-based, symptom-oriented treatment for breathlessness, such as fans or pursed lip breathing technique | 32(86.5) | 34(91.9) |
| Influencing my decision to review current strategies to manage breathlessness including inhaler use | 29(78.4) | 31(83.8) |
| Influencing my decision to refer person on for additional therapeutic or social services | 26(70.3) | 28(75.7) |
| Influencing my decision to intensify treatment of the patient's underlying condition | 21(56.8) | 24(64.9) |
| Influencing my decision to add/refer for pharmacologic-based, symptom-oriented treatment for breathlessness, such as opioids | 18(48.6) | 22(59.5) |
| Influencing my decision to pursue additional diagnostic testing | 16(43.2) | 13(35.1) |
| **Influencing my decision regarding timing of discharge (for hospitalised people)** | **10(27.0)** | **18(48.6)** |
|  | | |
| **Which of the following non-pharmacological/non-surgical therapies are effective for the relief of chronic breathlessness?** select all that apply | | |
| Pursed lip breathing | 36(97.3) | 37(100) |
| Pacing/fatigue management | 36(97.3) | 36(97.3) |
| Pulmonary rehabilitation/exercise training | 35(94.6) | 37(100) |
| Positioning to alleviate breathlessness | 34(91.9) | 37(100) |
| Relaxation techniques | 34(91.9) | 36(97.3) |
| Walking aids and home modification | 34(91.9) | 35(94.6) |
| Cool air/fan | 33(89.2) | 37(100) |
| Mindfulness techniques | 32(86.5) | 36(97.3) |
| **Cognitive behavioural strategies** | **30(81.1)** | **37(100)** |
| Non-invasive ventilation | 20(54.1) | 22(59.5) |
| Oxygen for non-hypoxaemic patients | 12(32.4) | 5(13.5) |

SOB=shortness of breath; bold type indicates significant difference in those who changed their view pre-post workshop according to McNemars test
