# Supplementary material for: Attitude change and increased confidence with management of chronic breathlessness following a health professional training workshop: a survey evaluation
Source: BMC Med Educ. 2020 Mar 30;20:90. doi: 10.1186/s12909-020-02006-7 (PMC7106669; doi:10.1186/s12909-020-02006-7)
Supplement: Supplementary file 4 — Additional file 4. Summarised free-text responses to post-workshop open questions about most and least helpful aspects, suggested changes and any other comments. [file 12909_2020_2006_MOESM4_ESM.docx]

**Additional File 4**: Summarised free-text responses to post-workshop open questions about most and least helpful aspects, suggested changes and any other comments.

| **Category of response** | Number of free text comments indicating this was the “most helpful” aspect of the workshop: total n=87 *(illustrative example comments in italics)*  **Related free text from “other comments” (number of comments)** ***(illustrative example comments in bold italics)*** | Number of free text responses indicating this was the “least helpful” aspect of the workshop: total n=20 *(illustrative example comments in italics)*  **Related free text from “suggested changes” (number of comments) *(illustrative example comments in bold italics)*** |
| --- | --- | --- |
| General positive comments | All aspects were helpful (6)  **General positive comments/gratitude (18)**  ***The best workshop by far that I have attended in > 30 years of working clinically*** | Nothing was least helpful (8)  **Nothing should be changed n=8**  ***No - I thought it was excellent especially as it was multi - disciplinary and relevant to palliative care.*** |
| Impact | **Specific impacts of course on clinical practice, changed thinking, recommendation to others (9)** |  |
| **Specific components of the course** | | |
| Conversations with people with breathlessness | Most helpful (13)  *Hearing from people living with breathlessness in non-clinician type interactions was good and better than just watching an edited video.* | Least helpful (9)  *I was a little uncomfortable during the carer guest discussion however that was possibly the point - definitely challenged my preconceived beliefs and comfort levels* |
| Printed resources | Most helpful (10)  *The Tool Kit presented to each participant (I am now using mine in my clinical practice).* | - |
| Psychological/thinking aspects of breathlessness including motivational interviewing and mindfulness | Most helpful (10)  *being confident to cause some breathlessness in my clients to then show how the experience can be better managed in future events by re-training the brain.*  *the content covered on Day 2 was exceptional and most useful - the information around the thinking component of breathlessness* | Least helpful (1)  *the pscyh input was useful however I felt that the one session probably wasn't enough to get a good understanding* |
| Content and evidence base on using the handheld fan and associated strategies | Most helpful (7)  *I had never heard of or used a fan before, so learning about that was useful* | - |
| Reflective and participatory sessions including explaining breathlessness | Most helpful (6)  *Also, as much as they are uncomfortable at the time the group work and 'elevator pitch' was fantastic to apply and consolidate knowledge*    *Practical sessions - practising how to explain breathlessness without jargon; practising answering patient questions/addressing concerns* | Least helpful (3)  *No practical demonstration of eg. inhaler technique, flutter pipe devices. It’s a long time since I was at uni and learnt about those things!*  *I am never a fan of role plays.*  **More stories/examples from people working in the area(2)**  **hands on training(1)**  **practical examples of assessments /interventions in action(3)**  **Less group work(1)** |
| Chronic pain research and parallel with chrionic breathlessness | Most helpful (5)  *Linking chronic breathlessness to chronic pain has really changed the way I view my management and patients. Very helpful.* | - |
| Understanding of the BTF clinical model | Most helpful (4)  Gaining an understanding of the cBIS model of care | - |
| Other course sessions | Most helpful:  Breathlessness; models and mechanisms (3)  Assessment of breathlessness (3)  Discussion/practical tips around management of breathlessness (2)  Reinforcement of current practice/understandings (2) | Least helpful:  Breathlessness; models and mechanisms (1)  Functioning component (1)  Question and answer session (1)  End of life issues (1)  Research information (1)  **Hear about other non-pharma interventions: NIV, high flow nasal cannula(1)**  **pharmacology: more time(2)**  **physiology(2)**  **more on pulmonary rehabilitation(1)** |
| **Interactions with other course participants** | | |
| Collaborations, discussions and networking | Most helpful (7)  *Collaboration and discussion with colleagues from different specialisations and areas of interest*  *I most appreciated the collegiality and shared learning (from multidisciplinary clinicians and from the 'patients' living with chronic breathlessness)*  **Great enthusiasm, group dynamics, open discussion (2)** | - |
| **Course organisation and delivery** | | |
| Pacing, structure , nature and organisation of content | Most helpful (6)  *Structure of course days was intuitive and at a pace that promoted learning*  *The way each of the breathing, thinking, functioning models were presented with strategies in each.*  **Well run and organised(4)** | Least helpful (2)  *I think that the group was too large and so some of the group work sessions were longer than they could have been with a smaller group.*  **Changes to session and course length (2)**  **Smaller group size(1)**  **Online delivery + weekend school/app/online course(3)**  **Take to other locations(1)** |
| Presenters positive qualities | Most helpful (3)  *The varied presentation styles and presenters*  **Presenters positive qualities (2)** | - |
| Access to speakers | Most helpful (3)  *Access to experts in field in an open positive environment* | - |
| Venue | Most helpful (1)  **Venue and catering excellent(2)** | **Improve slide size and (sound) system(1)** |
