# Supplementary material for: Attitude change and increased confidence with management of chronic breathlessness following a health professional training workshop: a survey evaluation
Source: BMC Med Educ. 2020 Mar 30;20:90. doi: 10.1186/s12909-020-02006-7 (PMC7106669; doi:10.1186/s12909-020-02006-7)
Supplement: Supplementary file 5 — Additional file 5. Potential barriers to implementation anticipated by the post-course respondents [file 12909_2020_2006_MOESM5_ESM.docx]

**Additional File 5:** Potential barriers to implementation anticipated by the post-course respondents (n=41).

| **Barrier Issue** | **Individual level** | **Clinical team/service level** |
| --- | --- | --- |
| Knowledge and beliefs(3) | Strategies to apply with people with dementia/cognitive impairment | Understanding the concept of chronic breathlessness |
|  |  | Broader team familiarisation with processes and understanding |
| Skills(3) | Need further training to implement strategies | Ability to review and reinforce skills and confidence in others (2) |
|  | Further time to teach strategies to myself e.g. mindfulness *“Advised of the Smiling Mind app so going to begin there”* |  |
| Resources: materials (7) | Development of really solid and user friendly resources is needed | I don’t have the resources to teach strategies to staff  *“Provided inservice on return…”* |
|  | Resources (unspecified) | |
|  | Accessing physical resources such as fans (4), permission to use resources | |
| Resources: prioritisation and time(10) | Sufficient time to assess chronic breathlessness including related psychological issues (3)  *“However, now feel better equipped to be more efficient and comprehensive”* | |
|  | Having other health professionals realise the importance to take the time to address this symptom | |
|  | Sufficient time to implement/reinforce strategies(3) | |
|  | Time (unspecified)(3) | |
| Resources: funding (3) | Funding unspecified (3) | |
| Organisational culture: social influence; staffing resources; professional role identity (10) | Challenge/effort required to start something new to the organisation (2)  *“I think I will try to start with applying the course learnings to individual patients and collect evidence that it is working. Start small..”* | |
|  | Slow to respond healthcare IT systems would hinder implementing new tools | |
|  | Workplace culture, time, resources disallowing professionals to apply all their knowledge | |
|  | Staffing by allied health not supported by management | |
|  | Hard to change staff behaviours | |
|  | Workload concerns of adding additional assessments/interventions | |
|  | Working within the resources of the system to ensure sustainability | |
|  | Provision of home-based service unlikely due to lack of funds/staffing | |
|  | Expanding the role of the nurse could be perceived as scope creep by allied health teams. | |

Multiple comments related to the same issue indicated by number in brackets. Comments that offered a planned or completed way to overcome the barrier are indicated in italics.
