# Supplementary material for: Attitude change and increased confidence with management of chronic breathlessness following a health professional training workshop: a survey evaluation
Source: BMC Med Educ. 2020 Mar 30;20:90. doi: 10.1186/s12909-020-02006-7 (PMC7106669; doi:10.1186/s12909-020-02006-7)
Supplement: Supplementary file 6 — Additional file 6. Exploratory factor analysis of items for familiarity, confidence and attitudes. [file 12909_2020_2006_MOESM6_ESM.docx]

**Additional File 6:** Exploratory factor analysis of items for familiarity, confidence and attitudes.

To explore the structure of familiarity (8 items), confidence (8 items) and attitudes (9 items) constructs, baseline data from participants was subjected to exploratory factor analysis of each construct using the statistical program SPSS (and confirmed with analysis robust to categorical variables using MPLUS in the case of the attitudes items). Regression factor scores were retained and Pearson correlations used to describe relationships within and between constructs of familiarity, confidence and attitudes.

One factor (Eigenvalue > 1) was identified as underlying the familiarity items and accounted for 68% of the variance in the questionnaire data.

Similarly, a single factor was identified as underlying the confidence items and accounted for 77% of the variance in the data.

As single factors were identified in these constructs, no rotation was applied.

An oblique rotation method (Direct oblimin) was selected in analysis of the attitudes items to allow for correlation between factors. Three factors (Table 1) were identified as underlying the nine items. In total these factors accounted for around 74% of the variance in the questionnaire data.

Table 1: Oblimin rotated factor structure of the 9-item attitudes section of the study questionnaire

| Item | Loadings | | |
| --- | --- | --- | --- |
|  | Factor 1 | Factor 2 | Factor 3 |
| 1. CB is one of the main symptoms that cause patients with advanced cardiopulmonary conditions (e.g. COPD, heart failure, interstitial lung disease) and cancer to seek medical care |  | 0.942 |  |
| 2. Relief of breathlessness is a central goal of the management of patients with advanced cardiopulmonary conditions/cancer. |  | 0.717 |  |
| 3. People who experience chronic breathlessness would like me to ask them about this symptom. | 0.830 |  |  |
| 4. People who experience chronic breathlessness are able to rate their own breathlessness intensity on a scale of 0-10. |  |  | 0.719 |
| 5. Serial measurements of breathlessness would be useful for assessing response to therapy. | 0.838 |  |  |
| 6. Breathlessness assessment by a scale should be part of the "vital signs" for patients with cardiopulmonary diseases. | 0.833 |  |  |
| 7. The person’s experience of chronic breathlessness should be used to guide treatment decisions independent of objective measures such as respiratory rate and oxygen saturation. |  |  | 0.744 |
| 8. Judicious use of oral and/or parenteral opioids can provide relief of chronic breathlessness |  |  | 0.803 |
| 9. Limited use of opioids for relief of chronic breathlessness in patients with advanced cardiopulmonary disorders is often due to concerns of respiratory depression. |  | 0.706 |  |
|  |  |  |  |
| Percentage of variance | 47% | 15% | 11% |
| Cronbach’s alpha of this factor | 0.832 | 0.834 | 0.705 |

CB=chronic breathlessness

Regression factor scores for familiarity and confidence were strongly correlated (r=0.965, p<0.001, Table 2). The strength of correlation between attitude factors was medium (0.4) but not significant between Factor 1 and 2. Regression factor scores for familiarity or confidence were not related to any of the three attitude factors in this analysis of baseline data (Table 2).

Table 2: Factor correlation matrix (Pearson’s r values, p<0.05 unless indicated)

|  | Familiarity | Confidence | Attitude Factor1 | Attitude Factor2 | Attitude Factor3 |
| --- | --- | --- | --- | --- | --- |
| Familiarity | 1.00 | 0.97 | 0.05# | 0.17# | -0.3# |
| Confidence |  | 1.00 | 0.03# | 0.12# | 0.1# |
| Attitude Factor1 |  |  | 1.00 | 0.29# | 0.41 |
| Attitude Factor2 |  |  |  | 1.00 | 0.36 |
| Attitude Factor3 |  |  |  |  | 1.00 |

#p≥0.05

This analysis suggests that at baseline, familiarity and confidence with the course objectives were closely related to each other, but attitudes measured by the questionnaire items were not related to familiarity/confidence with the objectives. A wide range of other factors not encapsulated by the familiarity/confidence items may be related to clinician attitudes regarding chronic breathlessness, for example, hope and optimism versus helplessness (1).

1. Macnaughton J, Carel H. Breathing and Breathlessness in Clinic and Culture: Using Critical Medical Humanities to Bridge an Epistemic Gap. In: Whitehead A, Woods A, Atkinson S, et al., editors. The Edinburgh Companion to the Critical Medical Humanities. Edinburgh (UK): Edinburgh University Press; 2016 Jun 30. Chapter 16. Available from: <https://www.ncbi.nlm.nih.gov/books/NBK379257/>. Accessed February 4 2020.
